# Specific Radioactivity of Neutron Induced Radioisotopes: Assessment Methods and Application for Medically Useful ^177^Lu Production as a Case

**DOI:** 10.3390/molecules16010818

**Published:** 2011-01-19

**Authors:** Van So Le

**Affiliations:** ANSTO Life Sciences, Australian Nuclear Science and Technology Organization, New Illawarra Road, Lucas Heights, P.M.B 1 Menai, NSW 2234, Australia; E-Mail: slv@ansto.gov.au; Tel.: +61297179725; Fax: +61297179262

**Keywords:** specific radioactivity, target burn-up, isotope dilution, neutron capture yield, nuclear reaction, nuclear reactor, radioisotope production, targeting radiopharmaceutical, ^177^Lu, ^175^Lu, ^176^Lu, ^177^Yb, ^176^Yb, ^175^Yb, ^174^Yb

## Abstract

The conventional reaction yield evaluation for radioisotope production is not sufficient to set up the optimal conditions for producing radionuclide products of the desired radiochemical quality. Alternatively, the specific radioactivity (SA) assessment, dealing with the relationship between the affecting factors and the inherent properties of the target and impurities, offers a way to optimally perform the irradiation for production of the best quality radioisotopes for various applications, especially for targeting radiopharmaceutical preparation. Neutron-capture characteristics, target impurity, side nuclear reactions, target burn-up and post-irradiation processing/cooling time are the main parameters affecting the SA of the radioisotope product. These parameters have been incorporated into the format of mathematical equations for the reaction yield and SA assessment. As a method demonstration, the SA assessment of ^177^Lu produced based on two different reactions, ^176^Lu (n,γ)^177^*Lu* and ^176^Yb (n,γ) ^177^Yb (β^-^ decay) ^177^Lu, were performed. The irradiation time required for achieving a maximum yield and maximum SA value was evaluated for production based on the ^176^Lu (n,γ)^17*7*^*Lu* reaction. The effect of several factors (such as elemental Lu and isotopic impurities) on the ^177^Lu SA degradation was evaluated for production based on the ^176^Yb (n,γ) ^177^Yb (β^-^ decay) ^177^Lu reaction. The method of SA assessment of a mixture of several radioactive sources was developed for the radioisotope produced in a reactor from different targets.

## 1. Introduction

State-of-the-art radiopharmaceutical development requires radioisotopes of specific radioactivity (SA) as high as possible to overcome the limitation of *in vivo* uptake of the entity of living cells for the peptide and/or monoclonal antibody based radiopharmaceuticals which are currently used in the molecular PET/CT imaging and endo-radiotherapy. The medical radioisotopes of reasonable short half-life are usually preferred because they have, as a rule of thumb, higher SA. These radioisotopes can be produced from cyclotrons, radionuclide generators and nuclear reactors. The advantage of the last one lies in its large production capacity, comfortable targetry and robustness in operation. This ensures the sustainable supply and production of key, medically useful radioisotopes such as ^99^Mo/^99m^Tc for diagnostic imaging and ^131^I, ^32^P, ^192^Ir and ^60^Co for radiotherapy. The high SA requirement for these radioisotopes was not critically considered with respect to their effective utilization in nuclear medicine, except for ^99^Mo. The current wide expansion of targeting endo-radiotherapy depends very much on the availability of high SA radionuclides which can be produced from nuclear research reactor such as ^153^Sm, ^188^W/^188^Re, ^90^Y and ^177^Lu. As an example, as high as 20 Ci per mg SA ^177^Lu is a prerequisite to formulate radiopharmaceuticals targeting tumors in different cancer treatments [1,2].

So far in radioisotope production, reaction yield has been the main parameter to be concerned with rather than SA assessment and unfortunately, the literature of detailed SA assessment is scarcely to be found [3,4]. The SA assessment of radioisotopes produced in a reactor neutron–activated target is a complex issue. This is due to the influence of the affecting factors such as target burn-up, reaction yield of expected radionuclide and unavoidable side-reactions. All these depend again on the available neutron fluxes and neutron spectrum, which are not always adequately recorded. Besides, the reactor power-on time and target self-shielding effect is usually poorly followed up. Certainly, the SA of target radionuclides has been a major concern for a long time, especially for the production of radioisotopes, such as ^60^Co and ^192^Ir, used in industry and radiotherapy. In spite of the target burn-up parameter present in the formula of reaction yield calculation to describe the impact of target depression, the SA assessment using the reaction yield was so significantly simplified that the target mass was assumed to be an invariable value during the reactor activation. Critically, this simplification was only favored by virtue of an inherent advantageous combination of the low neutron capture cross section (37 barns) of the target nuclide ^59^Co and the long half-life of ^60^Co (which keeps the amount of elemental Co unchanged during neutron bombardment) [4].

The targets used in the production of short-lived medical radioisotopes, however, have high neutron capture cross sections to obtain as high as possible SA values. This fact causes a high “real” burn-up of the target elemental content. Especially, the short half-life of the beta emitting radioisotope produced in the target hastens the chemical element transformation of the target nuclide and strongly affects the SA of the produced radioisotope. The triple factors influencing the production mentioned above (target, neutron flux and short half-life of produced radionuclide) are also critical with respect to the influence of the nuclear side-reactions and impurities present in the target. Moreover, the SA of a radionuclide produced in nuclear reactor varies with the irradiation and post-irradiation processing time as well. All these issues should be considered for a convincing SA assessment of the producible radioisotope for any state-of-the-art nuclear medicine application. As an example, a theoretical approach to the SA assessment reported together with an up-to-date application for ^177^Lu radioisotope production is presented in this paper. This assessment can also play a complementary or even substantial role in the quality management regarding certifying the SA of the product, when it may be experimentally unfeasible due to radiation protection and instrumentation difficulties in the practical measurement of very low elemental content in a small volume solution of high radioactivity content. 

High SA nuclides can be produced by (n, *γ*) reaction using high cross section targets such as the ^176^Lu (n, *γ*)^177^Lu reaction (б = 2,300 barns). ^177^Lu is a radioisotope of choice for endo-radiotherapy because of its favorable decay characteristics, such as a low energy beta decay of 497 keV (78.6%) and half-life of 6.71 day. It also emits gamma rays of 113 keV (6.4%) and 208 keV (11%) which make it useful for imaging in-vivo localization with a gamma camera.

^177^Lu can be produced by two different routes, a direct route with the ^176^Lu (n, *γ*)^177^Lu reaction and an indirect route via the ^176^Yb (n, *γ*) ^177^Yb (*β*^-^ decay) ^177^Lu nuclear reaction-transformation. The direct route could be successfully performed in high neutron flux nuclear reactors but these are available in only a handful of countries in the world. Additionally, large burn-up of the target nuclide during high neutron flux irradiation may cause a degradation of the SA value of the produced nuclide if the target contains isotopic impurities. No-carrier-added (n.c.a) radioisotopes of higher SA can be produced via an indirect route with a nuclear reaction- followed –by- radioactive transformation process, such as in the process of neutron capture-followed-by- *β*^-^ decay , ^176^Yb (n, *γ*) ^177^Yb (*β*^-^ decay) ^177^Lu. In this case, the same reduction in SA is also be experienced if the target contains isotopic and/or elemental Lu impurities.

^177^Lu production has been reported in many publications [5,6,7,8,9], but until now the product quality, especially the evaluation of ^177^Lu specific radioactivity in the product, has not been sufficiently analyzed. Based on the theoretical SA assessment results obtained in this report, the optimal conditions for the ^177^Lu production were set up to produce ^177^Lu product suitable for radiopharmaceutical preparations for targeting endo-radiotherapy. 

### 1.1. Units of specific radioactivity, their conversion and SA of carrier-free radionuclide

The specific radioactivity is defined by different ways. In our present paper we apply the percentage of the hot atom numbers of a specified radioactive isotope to the total atom numbers of its chemical element present in the product as the specific radioactivity. This is denoted as atom %. 

The following denotation will be used for further discussion. N_Ri(A)_ is the hot atom numbers of radioisotope R_i_ of the chemical element A and *λ_Ri_*_(*A*)_, its decay constant. N_A_ is the atom numbers of the chemical element A and T_1/2_ (sec) the half-life of radioisotope R_i_.

The SA unit of atom % is defined as follows: 

$SA\;(in\;unit\;atom\;\%)=\frac{100\times Hot\;atom\;numbers\;of\;a\;specified\;radionuclide}{Atom\;numbers\;of\;the\;chemical\;element\;of\;specified\;radionuclide}$ which can be formulated as follows:

(1) $$SA\;(atom\;\%)=100\cdot N_{Ri(A)\;}/N_{A}$$

SA in units Bq /Mol and Bq/g are more currently used in practice. The conversion between the SA units is the following:
(2)
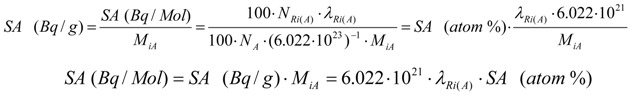

where M_iA_ is the atomic weight of the target or radioactive material of given isotopic composition of the chemical element A. 

For a radioactive material containing n isotopes of the element A:

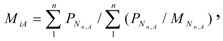

where $P_{N_{n,A}}$ and $M_{N_{n,A}}$ are the weight percentage and atomic weight of the isotope N_n,A_, respectively. The specific radioactivity of the carrier-free radioisotope R_i_ is calculated as below:
(3)
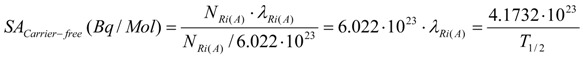


Identifying eq.2 with eq.3 (individualizing M_iA_ as the atomic weight of the concerned radioisotope), it is clear that the SA of a carrier-free radionuclide in unit atom % is 100%.

## 2. Theoretical Approach and Assessment Methods

Reactor-based radioisotope preparation usually involves two main nuclear reactions. The first one is the thermal neutron capture (n, *γ*) reaction. This reaction doesn’t lead to a radioisotope of another chemical element, but the following radioactive *β*^−^ decay of this isotope during target activation results in a decrease in both the reaction yield and atom numbers of the target chemical element. The second reaction is the thermal neutron capture followed by radioactive transformation S (n, *γ*) R_x_ (*β*^−^ decay) R_i_. This reaction leads to a carrier-free radioisotope of another chemical element than the target chemical element. 

The SA assessment in the radioisotope production using the first reaction (with a simple target system) is simple. Careful targetry could avoid the side reaction S (n, *γ*) R_x_ (*β*^−^ decay) R_i_ which could result in the isotopic impurities for the radioisotope intended to be produced using the first reaction. In this case the SA assessment in (n, *γ*) reaction based production process can be simplified by investigation of the SA degrading effect of target nuclide burn-up, chemical element depression due to radioactive decay and isotopic impurities present in the target.

On the other hand the SA assessment in the radioisotope production using the second reaction (with complex target system) is more complicated. The complexity of the targetry used in S (n, *γ*) R_x_ (*β*^-^ decay) R_i_ reaction based isotope production requires an analysis of the combined reaction system. This system is influenced by both (n, *γ*) reaction and neutron-capture- followed-by-radioactive transformation S (n, *γ*) R_x_ (*β*^-^ decay) R_i_. So the effect of side nuclear reactions in this target system will be assessed in addition to the three above mentioned factors that are involved in the simple target system. In this case the SA assessment is best resolved by a method of SA calculation used for the mixture of several radioactive sources of variable SA, which is referred to as a radioisotope dilution process.

For the calculation of SA and reaction yield of the radioisotope R_i_ in the two above mentioned reactions, the following reaction schemes are used for further discussion.

Reaction scheme 1:

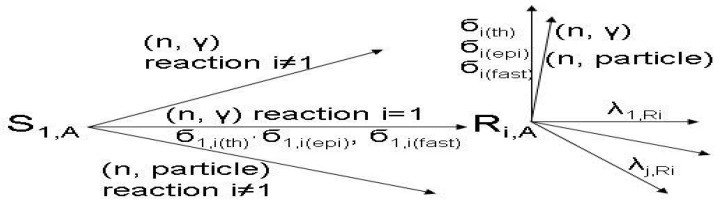



Reaction scheme 2:

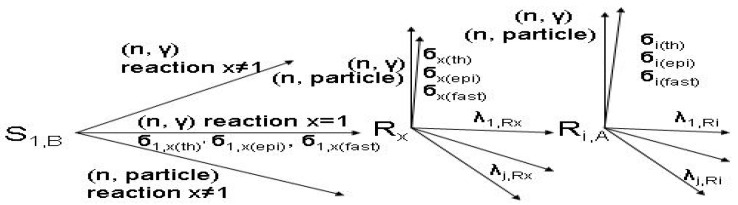



Reaction scheme 3:

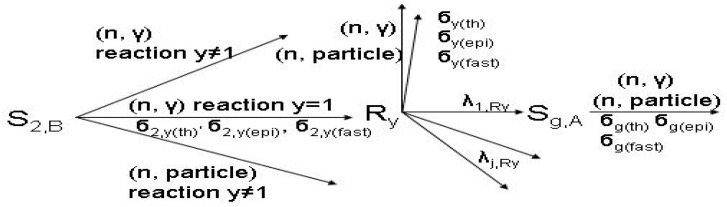

S_1,A_ is the target stable isotope of element A in the target; S_g,A_ (with g ≥ 2) is the impure stable isotope of element A originally presented or produced in the target.
S_1,B_ is the target stable isotope of element B in the target; S_2,B_ is the stable isotope of element B in the target.
R_i,A_ or R_i_ is the wanted radioisotope of element A produced in the target from stable isotope S_1,A_.
R_x_ and R_y_ are the radioisotopes of element B produced in the target.
The particle emitted from reaction (n, particle) may be proton or alpha.
σ_(th)_, σ_(epi)_ and σ_(fast)_ are reaction cross sections for thermal, epi-thermal and fast neutrons, respectively.
σ_1,i(th)_, σ_2,x(th)_, σ_2,y(th)_,. are cross sections of thermal neutrons for the formation of isotopes i, x, y,. from stable isotope 1, 2, 2, respectively.
λ is the decay constant.

The (n, *γ*) reaction yield and the specific radioactivity calculated from it depends on the neutron flux and reaction cross-section which is variable with neutron energy (*E_n_*) or velocity (*v_n_*). In the thermal neutron region, the cross-section usually varies linearly as 1/*v_n_* (so called 1/*v_n_* reaction), where *v_n_* is velocity of neutrons. The cross section-versus-velocity function of many nuclides is, however, not linear as 1/*v_n_* in the thermal region (so called *non* − 1/*v_n_* reaction.). As the energy of neutrons increases to the epithermal region, the cross section shows a sharp variation with energy, with discrete sharp peaks called resonance. 

On other hand, the cross section values of the (n, *γ*) reactions tabulated in the literature present as σ_0_ given for thermal neutrons of *E_n_* = 0.0253 *eV* and *v_n_* = 2200 *m*/*s* and as *I*_0_ (infinite dilution resonance integral in the neutron energy region from E_Cd_ = 0.55 eV to 1.0 MeV) given for epithermal neutrons.

The symbols *σ_th_* and *σ_epi_* used in this paper are identified with the thermal neutron activation cross-section *σ*_0_ and the infinite dilution resonance integral *I*_0_, respectively, for the case of 1/*v_n_* (n,*γ*)-reaction carried out with a neutron source of pure 1/*E_n_* epithermal neutron spectrum (Epithermal flux distribution parameter *α* = 0). Unfortunately, this condition is not useful any more for practical reaction yield and SA calculations.

In practice the target is irradiated by reactor neutrons of $1/E_{n}^{1+A}$ epithermal neutron spectrum, so the value of Ω*_i_* presenting as a sum *σ*_1,*i*(*th*)_ + *R_epi._σ*_1,*i*(*epi*)_ in all the equations below has to be replaced by *σ_eff_*_(*1/v*)_ for the “1/*v_n_”*- named (n,γ) reaction and by *σ_eff_*_(non − *1/v*)_ for the “non − 1/*v_n_*” - named (n,γ)- reaction. The detailed description of these *σ_eff_* values can be found in the ‘Notes on Formalism’ at the end of this section. 

For the isotope production based on (n,γ) reactions the neutron bombardment is normally carried out in a well-moderated nuclear reactor where the thermal and epithermal neutrons are dominant. The fast neutron flux is insignificant compared to thermal and epithermal flux (e.g. <10^7^ n.cm^−2^.s^−1^ fast neutron flux compared to >10^14^ n.cm^−2^.s^−1^ thermal one in the Rigs LE7-01 and HF-01 of OPAL reactor-Australia). Besides, the milli-barn cross-section of (n,γ) ,(n,p) and (n,α) reactions induced by fast neutrons is negligible compared to that of (n,γ) reaction with thermal neutron [11]. So the reaction rate of the fast neutron reactions is negligible. Nevertheless, for the generalization purposes the contribution of the fast neutron reaction is also included in the calculation methods below described. It can be ignored in the practical application of SA assessment without significant error. 

### 2.1. The specific radioactivity of radionuclide R_i_ in the simple target system for the (n, γ) reaction based radioisotope production

#### 2.1.1. Main characteristics of the simple target system

The simple target system contains several isotopes of the same chemical element. Among them only one radioisotope R_i_ is intended to be produced from stable isotope S_1,A_ via a (n, *γ*) reaction i = 1 as described above in reaction scheme 1. Other stable S_g,A_ isotopes ( with g ≥ 2) of the target are considered as impure isotopes.

##### 2.1.1.1. The target burn-up for each isotope in simple target system 

The burn-up of the isotope S_1,A_ is the sum of the burn-up caused by different (n,γ) and (n, particle) reactions from reaction i = 1 to i = k, the cross sections of which are different б_1,i_ values. This total burn up rate could be formulated as follows: (4)
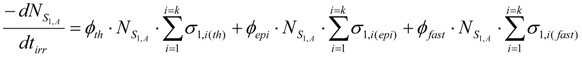

*σ*_1,*i*(*th*)_, *σ*_1,*i*(*epi*)_ and *σ*_1,*i*(*fast*)_ are the thermal, epithermal and fast neutron cross section of the S_1,i_ nuclide for the reaction i, respectively. *ϕ_th_*, *ϕ_epi_* and *ϕ_fast_* are the thermal, epithermal and fast neutron flux, respectively. t_irr_ is the irradiation time. $N_{S_{1,A}}$ is the atom numbers of the isotope S_1,A_. By putting *R_epi_ = ϕ_epi_/ϕ_th_* and *R_fast_ = ϕ_fast_/ϕ_th_* ratios into eq.4, the following is deduced. 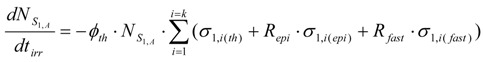


By substituting:(5)
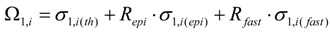

and:(6)
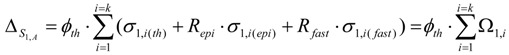

the above differential equation is simplified as follows:(7)
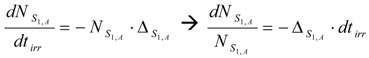


The un-burned atom numbers of the isotope S_1,A_ at any t_irr_ values ($N_{S_{1,A}}$) is achieved by the integration of eq.7 with the condition of $N_{S_{1,A}}=N_{0,}{}_{S_{1,A}}$ at t_irr_ = 0. The result is: (8)
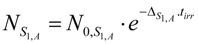

From this equation, the burned-up atom numbers of the isotope S_1,A_ ($N_{b,}{}_{S_{1,A}}$) is: (9)
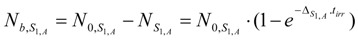

The same calculation process is performed for any isotope S_g,A_.

*Half-burn-up time of the target nuclide.* At half-burn-up time T_1/2-B_ a half of the original atom numbers of the isotope S_1,A_ are burned. Putting $N_{S_{1,A}}=N_{0,}{}_{S_{1,A}}/2$ into eq. 8, the T_1/2-B_ value is achieved as follows:(10)
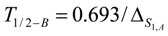


###### 2.1.1.2. Reaction yield of radioisotope R_i_ in the simple target system

By taking into consideration the un-burned atom numbers of the isotope S_1,A_ (eq. 8) , the reaction rate of any isotope in reaction scheme 1 will be evaluated as follows. In this reaction process the depression of the atom numbers of radioisotope R_i_ is caused by beta radioactive decays and (n, γ)/(n, particle) reaction-related destruction. The depression factor $\Lambda_{R_{i}}$ of the radioisotope R_i_ in reaction scheme 1 is formulated as follows: (11)
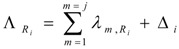

where Δ*_i_* = *ϕ_th_*·ΣΩ*_i_* and Ω*_i_* = σ_*i*(*th*)_ + *R_epi_*·*σ*_*i*(*epi*)_ + *R_fast_*·*σ*_*i*(*fast*)_

Taking into account eq.5, R_i_ radioisotope formation rate is the following:(12)
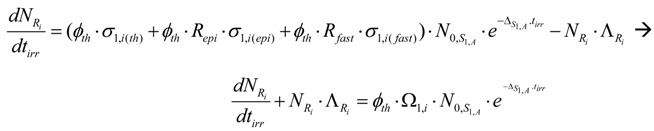


By multiplying both sides of this equation with $e^{\Lambda_{Ri}\cdot t_{irr}}$ and manipulating with the mathematical tool $\frac{d(XY)}{dt}=X\prime \cdot Y+X\cdot Y\prime$, this equation is converted into the following form:
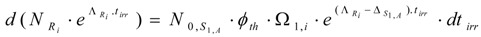


By integrating this equation and assuming N_Ri_ = 0 at t_irr_ = 0, the yield of radioisotope R_i_ at the irradiation time t_irr_ is the following:

The R_i_ atom numbers (N_Ri_):(13)
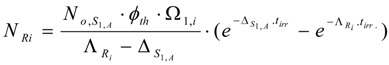


The R_i_ isotope radioactivity (A_Ri_):(14)
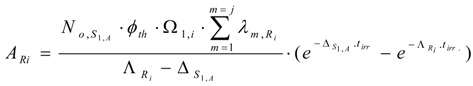


These equations can be deduced from the well known Bateman equation [3,12]. The R_i_ atom numbers and radioactivity at the post-irradiation time t_c_ ( N_Ri,tc_ and A_Ri,tc_, respectively) are calculated by multiplying eqs.13 and 14 with the factor $e^{-t_{c}\cdot \sum_{m=1}^{m=j}\lambda_{m,Ri}}$. 

*Maximum yield of radioisotope R_i_*. At the irradiation time (denoted as t_irr-max_) where $\frac{dA_{Ri}}{dt_{irr}}=0$, R_i_ radioactivity reaches maximum (*A_Ri_*_−max_). By differentiating eq.14 and making it equal to zero: 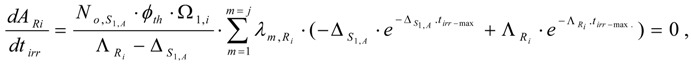

the *t_irr-max_ is* deduced as follows:(15)
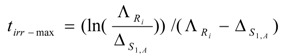


Equation (15) is useful for irradiation optimization to produce R_i_ radioisotope of highest yield. By introducing the value t_irr-max_ into eqs.13 and 14, we achieve the maximum yield of radioisotope R_i_ ( N_Ri-max_ and A_Ri-max_) as follows: 

The maximum atom numbers N_Ri-max_ is:(16)
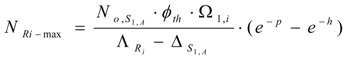


The maximum radioactivity A_Ri-max_ is: (17)
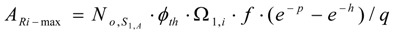

where $D=(\frac{\Lambda_{Ri}}{\Delta_{S_{1,A}}});\;f=(\sum_{m=1}^{m=j}\lambda_{m,Ri})/\Lambda_{Ri};\;p=\frac{\mathrm{In}\;D}{\left(D-1\right)};\;h=D\cdot \frac{\mathrm{In}\;D}{\left(D-1\right)};\;q={\left(1-D\right)}^{-1}$

As shown the maximum yield of radioisotope R_i_ is a function of the variable D.

#### 2.1.2. The SA assessment of radionuclide R_i_ in the simple target for (n, *γ*) reaction based radioisotope production

##### 2.1.2.1. General formula of SA calculation for the simple multi-isotope target

The simplification in the calculation is based on the fact that the target isotope S_i,A_ captures neutrons to form the wanted radioisotope R_i_ and the isotopic impurities in the target don’t get involved in any nuclear reactions whatsoever. The isotopic impurities may participate in some nuclear reactions to generate either stable isotopes of the target element or an insignificant amount of the isotopes of other chemical element than the target one. This simplified calculation process is supported by a careful targetry study regarding minimizing the radioactive isotopic impurities in the radioisotope product. The following is the SA of radioisotope R_i_ formed in a target composed of different stable isotopes: (18)
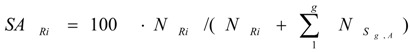

$\sum_{1}^{g}N_{S_{g,A}}$ is the sum of the remaining (unburned) atom numbers of g different stable isotopes of the same chemical element in the target. By placing the values $N_{S_{g,A}}$ of different stable isotopes of the target from eq.8 into this equation, the following general formula is obtained for the SA of radioisotope R_i_: (19)
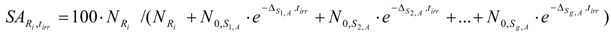

where $N_{0,S_{g,A}}=6.02\cdot 10^{23}\cdot m\cdot P_{g}/(100\cdot M_{g});\;N_{0,S_{g,A}}=6.02\cdot 10^{23}\cdot m\cdot P_{1}/(100\cdot M_{1})$

If the target contains impure isotopes of another chemical element, more stable isotopes of chemical element A generated via reaction scheme 3 above could be present in the denominator of this formula. This amount may cause additional depression of $SA_{R_{i},t_{irr}}$. This small impurity will, however, bring about an insignificant amount of stable isotope S_g,A_ and its depression effect will be ignored.

The eq.19 is set up with an ignorance of insignificant amount of not-really-burned impure stable isotope which captures neutron, but not yet transformed into the isotope of other chemical element via a radioactive decay).

If the impure isotope S_g,A_ doesn’t participate in any nuclear reaction or its neutron capture generates a stable isotope of the target element, then zero value will be given to the parameter Δ*S_g_*_,*A*_ of eq.(19). 

##### 2.1.2.2. SA of radioisotope R_i_ in the simple two-isotope target 

From the practical point of view, the target composed of two stable isotopes is among the widely used ones for radioisotope production. For this case the SA calculation is performed as follows:(20)
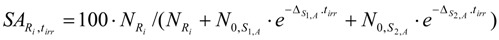

where $N_{0,S_{1,A}}=6.02\cdot 10^{23}\cdot m\cdot P_{1}/(100\cdot M_{1});\;N_{0,S_{2,A}}=6.02\cdot 10^{23}\cdot m\cdot P_{2}/(100\cdot M_{2})$, R_i_ is the radioisotope expected to be produced from the stable isotope S_1,A_ . P_1_ and *M*_1_, the weight percentage and atomic weight of the isotope S_1,A_, respectively. P_2_ and *M*_2_ are for the isotope S_2,A_, m is the weight of the target. 

By replacing $N_{0,S_{1,A}}$, $N_{0,S_{2,A}}$ and the $N_{R_{i}}$ value from eq.(13) into eq.(20), SA of radioisotope R_i_ in a two isotope target at the end of neutron bombardment, $SA_{R_{i},t_{irr}}$, is the following:(21)
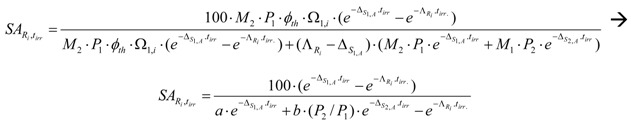

where $a=[\frac{\phi_{th}\cdot \Omega_{1,i}+\Lambda_{R_{i}}-\Delta_{S_{1,A}}}{\phi_{th}\cdot \Omega_{1,i}}];\;b=\frac{M_{1}\cdot (\Lambda_{R_{i}})-\Delta_{S_{1,A}}}{M_{2}\cdot \phi_{th}\cdot \Omega_{1,i}}$

SA at the post- bombardment time t_c_, $SA_{R_{i},t_{c}}$, is:(22)
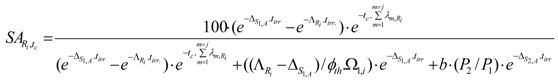


*Maximum SA of radioisotope R_i_ in the simple two-isotope target.* Rendering the differential of eq. 21 equal to zero offers the way to calculate the irradiation time at which the SA of nuclide R_i_ reaches maximum value ($SA_{R_{i},\max}$):(23)
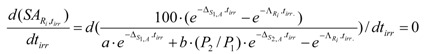


The irradiation time where the SA reaches maximum is denoted as $t_{irr,SA_{\max}}$. The equation for the calculation of the $t_{irr,SA_{\max}}$ value, which is derived from the above differential equation, is the following: (24)
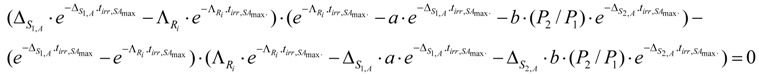


The solution of this equation performed by the computer software MAPLE-10 ^x^ gives the value $t_{irr,SA_{\max}}$. The analysis of the equation 24 and MAPLE-10 calculation results confirmed that the SA of nuclide R_i_ reaches maximum at a defined characteristic irradiation time $t_{irr,SA_{\max}}$ except for the case of P_2_=0 or very large $\Delta_{S_{2,A}}$ value, which will be investigated in the following sections. 

*SA of radioisotope R_i_ in the simple two-isotope target at the maximum reaction yield.* Replacing t_irr_ of eqs.(21) and (22) with the *t_irr-max_* from eq.15 is to calculate SA at the maximum reaction yield $SA_{R_{i},t_{irr,\max}}$ (achieved at the irradiation time *t_irr-max_*): (25)
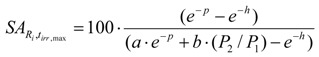

where $a=[\frac{\phi_{th}\cdot \Omega_{1,i}+\Lambda_{R_{i}}-\Delta_{S_{1,A}}}{\phi_{th}\cdot \Omega_{1,i}}];\;b=\frac{M_{1}\cdot (\Lambda_{R_{i}})-\Delta_{S_{1,A}}}{M_{2}\cdot \phi_{th}\cdot \Omega_{1,i}};\;D=(\frac{\Lambda_{R_{i}}}{\Delta_{S_{1,A}}});\;p=\frac{\mathrm{In}\;D}{\left(D-1\right)};\;h=D\cdot \frac{\mathrm{In}\;D}{\left(D-1\right)}$

These parameters are identical to that of the eq.(17) and (21).

*SA of radioisotope R_i_ in the target which is considered as a simple two-isotope target.* It is also a matter of fact that another very commonly used target system contains more than two stable isotopes (simple multi-isotope target system, *g ≥ 2)*. Except S_1,A_ as shown in reaction scheme 1, all the impure isotopes of the same chemical element in the target don’t get involved in any nuclear reactions or they may participate in with very low rate giving insignificant burn-up ($\Delta_{S_{g,A}}=0$*).*This system is considered as a special two-isotope target system for which the non-depression of impure isotopes ($\Delta_{S_{g,A}}=0$) and the combined impure isotope percentage (*P_imp_*_,*A*_) and molecular weight (*M_imp_*_,*A*_) are applied. We will have the relevant equations for the calculation of the specific SA value of this target by putting $\Delta_{S_{2,A}}=\Delta_{S_{g,A}}=0$, $M_{2}=M_{imp,A}=(\sum_{2}^{g}P_{S_{g,A}})/\sum_{2}^{s}(P_{S_{g.A}}/M_{S_{g.A}})$ *and* $P_{2}=P_{imp,A}=\sum_{2}^{g}P_{S_{g,A}}$ into eqs. (21)-(25) ($P_{S_{g,A}}$ and $M_{S_{g,A}}$ are the weight percentage and atomic weight of impure stable isotopes S_g,A_, respectively).

##### 2.1.2.3. SA of radioisotope R_i_ in the simple one-isotope target system

By introducing P_2_ = 0 into eq.(21), the SA of radioisotope R_i_ in the simple one-isotope target is the following:(26)
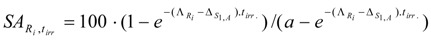


This equation doesn’t give the maximum value of $SA_{R_{i},t_{irr}}$.The result is confirmed by a calculation with MAPLE 10 software. A double check is made by putting the differential of eq. (26) equal to zero to investigate whether a maximum SA could be found: (27)
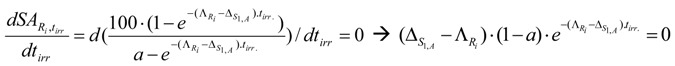


It is shown that the achieved differential eq. (27) has no solution with the variable t_irr_ and gives a correct solution when t_irr_ value approaches to infinity .When $(\Delta_{S_{1,A}}-\Lambda_{R_{i}})=0$ then a = 1 (as shown in eq.25), hence the differential value is not defined, so the specific radioactivity has no maximum value at any time. This means that the SA of nuclide R_i_ in the stable isotope target of 100% isotopic purity never reaches maximum at any irradiation time. 

It is also worth mentioning that when the value of $\Delta_{S_{2,A}}$or $\Delta_{S_{g,A}}$is very large, eq. (21) is converted to eq. (26). It means that the high burn-up of impure stable isotope S_g,A_ makes a multi-isotope target system change to a one-isotope target one. So, no maximum SA will be expected with this type of multi-isotope target system too.

As shown in eq. (26) the SA of these target systems increases with t_irr_. This fact teaches us that a compromise between maximum yield achievable at t_irr,max_ and favorable higher SA at the time t_irr>_ t_irr,max_ is subject to the priority of the producer.

### 2.2. The specific radioactivity of radionuclide R_i_ in a complex target system for the S(n, γ) R_x_ (β^-^ decay) R_i_ reaction based radioisotope production

#### 2.2.1. Main characteristics of the complex target system 

The complex target system contains several isotopes of different chemical elements. Among them only one radioisotope R_i_ is intended to be produced from stable isotope S_1,B_ of chemical element B via a S_1,B_ (n, *γ*) R_x_ (*β*^-^ decay) R_i_ reaction i = 1 as described above in reaction scheme 2. Other stable S_g,B_ isotopes ( with g ≥ 2) of the element B are considered as impure isotopes and they could be transformed into other isotopes (except R_i_ ) of the chemical element A as described above in reaction scheme 3. Besides, the target could contain different isotopes of the element A as impure isotopes which could be involved in different nuclear reactions during target irradiation.

##### 2.2.1.1. The yield of S_1B_(n, *γ*) R_x_ (*β*^-^ decay) R_i_ reaction 

This reaction generates a carrier-free radioisotope *R_i_* . The SA_Ri_ value is 100 atom %. As shown in reaction scheme 2, the atom numbers (N_Ri_) and the radioactivity (A_Ri_) of R_i_ radioisotope of chemical element A are calculated based on the general Bateman equation[3,12]. This is detailed in the following equation:(28)
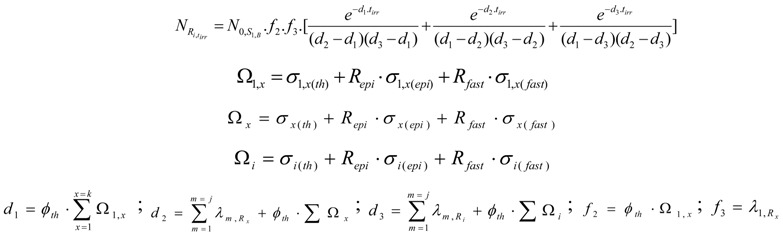


The R_i_ atom numbers $N_{R_{i,t_{c}}}$ present at post-irradiation time t_c_ is achieved by multiplying the $N_{R_{i,t_{irr}}}$ value with the decay factor $e^{-\sum_{m=1}^{m=j}\lambda_{m,R_{i\cdot t_{c}}}}$.

The A_Ri_ value is simply derived by multiplying the N_Ri_ values with $\sum_{m=1}^{m=j}\lambda_{m,R_{i}}$

##### 2.2.1.2. SA-degradation effect of impure stable isotope generated from S_2,B_(n, *γ*) R_y_(*β*^-^ decay) S_g_,_A_reaction 

Referred to reaction scheme 3 involving the impure stable isotope S_2,B_ in the S_1,B_ target , reaction S_2,B_(n, *γ*) R_y_ (*β*^-^ decay) S_g,A_ generates an amount of stable isotope S_g,A_ of the same chemical element to the wanted radionuclide R_i,A_. This fact makes the SA of radionuclide R_i,A_ produced from stable isotope S_1,B_ lower, so the atom numbers of the stable isotope S_g,A_ should be evaluated for the purpose of SA assessment. The atom numbers of S_g,A_ is determined based on the activity of radioisotope R_y_. Identifying eqs. (13) and (14) described for reaction scheme 1 with the process of reaction scheme 3, we get the following equations.

The atom numbers ($N_{R_{y},t_{irr}}$) and the radioactivity ($A_{R_{y},t_{irr}}$) of radionuclide R_y_ at irradiation time t_irr_ are calculated in the same manner as in Section 2.1.1.2 above (using eqs. (13) and (14)):(29)
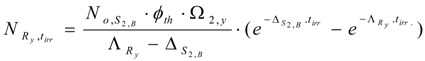

(30)
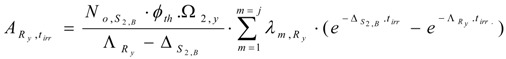

$\Delta_{S_{2,B}}$ is for stable isotope S_2,B_ and $\Delta_{S_{2,B}}=\phi_{th}\cdot \sum_{y=1}^{y=k}\Omega_{2,y}$

where $\Omega_{2,y}=\sigma_{2,y(th)}+R_{epi}\cdot \sigma_{2,y(epi)}+R_{fAst}\cdot \sigma_{2,y(fAst)}$

$\Lambda_{R_{y}}$ for radionuclide R_y_ and $\Lambda_{R_{y}}=\sum_{m=1}^{m=j}\lambda_{m,R_{y}}+\Delta_{Ry}$, where $\Delta_{R_{y}}=\phi_{th}\cdot \sum \Omega_{R_{y}}$

The partial radioactivity of radionuclide R_y_ for the formation of S_g,A_ isotope is denoted as $A_{R_{y}}\to S_{g,A},t_{irr}$. This quantity is calculated by either multiplying eq. (30) with a branch decay ratio $f_{S_{g,A}}$ or using an individual decay constant $\lambda_{R_{y}}\to S_{g,A}$ as follows: (31)
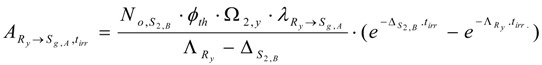


The S_g,A_ content ($N_{S_{g,A},t_{irr}}$) formed during neutron activation of the impure stable isotope S_2,B_ is calculated by integrating R_y_ nuclide radioactivity for the neutron irradiation time t_irr_ as below.
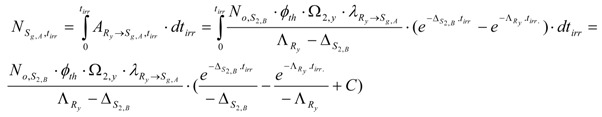


At t_irr_ = 0, $N_{S_{g,A},t_{irr}}=0$, then $C=\frac{\Lambda_{R_{y}}-\Delta_{S_{2,g}}}{\Delta_{S_{2,g}}\cdot \Lambda_{R_{y}}}$. Putting C value into the above equation we get:(32)
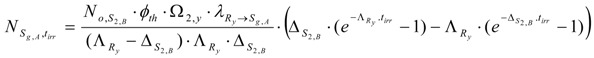


At post-irradiation time (t_c_) R_y_ radioactivity decreases as below:(33a)
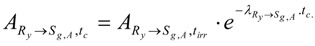


The radioactivity $A_{R_{y}}\to S_{g,A},t_{c}$ is equal to the formation rate of S_g,A_ during decay time. By integrating this formation rate we get the S_g,A_ atom numbers ($N_{S_{g,A},t_{c}}$) collected at the time t_c_. Because the $A_{R_{y}}\to S_{g,A},t_{irr}$ of nuclide R_y_ at the end-of- neutron-bombardment (E.O.B) time t_irr_ is independent on the variable t_c_, we get the following integral: 
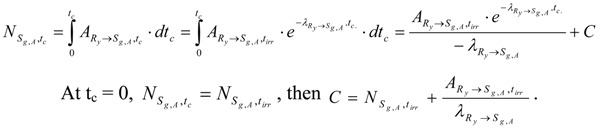


Putting the C value into the above equation we have:
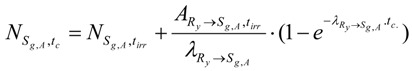


Further putting the $A_{R_{y}}\to S_{g,A},t_{irr}$ value of eq. (31) and $N_{S_{g,A},t_{irr}}$ of eq. (32) into this equation, we get the result below:(33b)
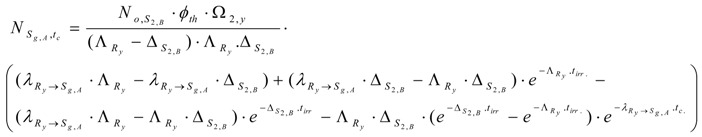


As shown in this equation, the S_g,A_ content ($N_{S_{g,A}}$) formed in the target from the S_2,B_ impure stable isotope is composed of a partial amount formed during neutron activation ($N_{S_{g,A},t_{irr}}$) of isotope S_g,A_ and its another part formed during post-irradiation decay of R_y_ induced in the target. 

The SA of R_i_ in the above mentioned target containing both S_1,B_ and S_2,B_ (as described in reaction schemes 2 and 3) is calculated using eqs. (28) and (33b) as follows: (34)
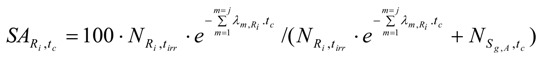


As a result of the analysis of the above equations, minimizing post-irradiation cooling/processing time is recommended to reduce the SA-degradation effect of the impure isotopes.

##### 2.2.1.3. SA-degradation effect of impure isotopes of the chemical element A 

The assessment of SA in system containing these impure isotopes can be found in the Section 2.1 for the simple target system.

#### 2.2.2. The SA assessment of radionuclide R_i_ in a complex target system

The radioisotope dilution is involved in SA depression in a complex target system in which both the wanted radioisotope R_i_ and its unfavorable stable isotope are generated from different nuclear reactions of both the target isotope and impurities. The complex target system is considered as a mixture of several radioactive sources of variable SA. The method of SA assessment for this mixture is formulated as below. 

SA_j,Ri_ is the SA of R_i_ in the radioactive source S_j_ the R_i_ radioactivity of which is A_j,Ri_ .The radioactive source S_j_ is produced in the target from a given nuclear reaction such as S (n, *γ*) R_x_ (*β*^-^ decay) R_i_ reaction (reaction scheme 2) or (n, *γ*) reaction (reaction scheme 1). There are n different radioactive sources S_j_ (j=1…n) in the target. So the target is a mixture of radioactive sources. The SA of this radioactive source mixture (SA_Mix,Ri_) is calculated as follows:(35)
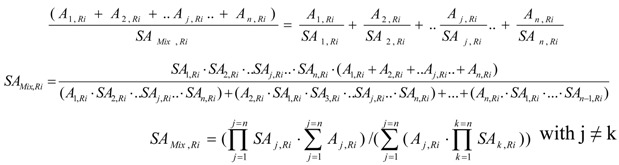

where $SA_{Mix,Ri}$ is in unit of atom %. $A_{j,Ri}$ is either the hot R_i_ atom numbers or R_i_ radioactivity of the relevant radioactive source j.

This equation is valuable for all values of SA, except SA_j,Ri_ = 0. This situation excludes the unfavorable effect of some (n, *γ*) reaction which generates a stable brother isotope S_g,A_ of radioisotope R_i_ in the target system ( Reaction scheme 3). To solve this problem we have to combine the atom numbers of this stable brother isotope with the atom numbers of one specified radioactive source of the mixture to generate a new radioactive source of SA ≠ 0, e.g. the combination of radioactive sources produced from the reactions in the scheme 2 and 3. This treatment will be detailed in a practical application for the ^176^Yb target system in the following section.

### 2.3. Notes on formalism

During neutron bombardment of the target in the nuclear reactor of $1/E_{n}^{1+A}$ epithermal neutron spectrum, the rate of (n,γ) reactions is calculated based on either Westcott or Hogdahl formalism [13] depending on the excitation function of the target nuclide ( the dependence of the reaction cross section on the neutron energy). 

For the “*non* − 1/*v_n_*” - named (n,γ) reactions the modified Westcott formalism can be used to improve the accuracy of reaction yield calculation and the reaction rate in both thermal and epithermal neutron region for a diluted sample (both the thermal and epithermal neutron self-shielding factors are set equal to 1 or very close to unity) is:(N.1)
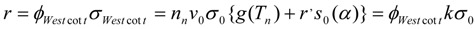

(where $\phi_{West\;\cot\;t}=n_{n}v_{0}$, k-factor *k* = {*g*(*T_n_*) + *r*’*S*_0_(*α*} and $\phi_{West\;\cot\;t}=k\sigma_{0}$).

In case that Westcott’s factor *g*(*T_n_*) = 1 the Westcott values are $\phi_{West\;\cot\;t}=n_{n}v_{0}=\phi_{th}\{1+f_{H}\xi (\alpha )\}$ and $\sigma_{West\;\cot\;t}=\sigma_{0}\{1+r\prime S_{0}(\alpha )\}$.Under this condition, the reaction rate is: (N.2)
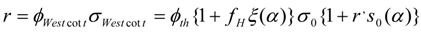


Westcott did not define a thermal neutron flux, but only a conventional (total) neutron flux was mentioned instead. It is the ascertainment from Westcott and Hogdahl formalism that: 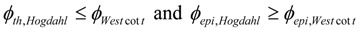


Practically, the values $\phi_{th,HogdAhl}$ and $\phi_{epi,HogdAhl}$ (usually known as thermal neutron flux $\phi_{th}$ and epi-thermal neutron flux $\phi_{epi}$, respectively) are determined by a specified nuclide (so called monitor) reaction based measurement of neutron flux in the spectral area below and above the cadmium cut-off energy (E_Cd_ = 0.55 eV), respectively, while the Westcott neutron flux values $\phi_{West\;\cot t}$ and $\phi_{epi,West\;\cot\;t}$ are not usually available. 

For the evaluation of the practical value of $\phi_{th,HogdAhl}$ versus $\phi_{West\;\cot t}$, the ratio $\left(\phi_{West\;\cot\;t}/\phi_{th,HogdAhl})=\{1+f_{H}\xi (\alpha )\right\}$ was calculated using the extreme values of epithermal flux distribution parameter *α*, the neutron energy E_0_ = 0.0253 eV and E_Cd_ = 0.55 eV and the Hogdahl ratio f_H_ = 0.02 (Rig LE7-01 of Australian OPAL reactor).

The values of ($\phi_{West\;\cot\;t}/\phi_{th,HogdAhl}$) ratio of 1.011 for *α* = −0.15 and 1.006 for *α* = 0.3 were achieved. These results suggest that the “*non* − 1/*v_n_*” - named (n,γ) reaction yield will be around 1% less than the real value if $\phi_{West\;\cot\;t}=\phi_{th,HogdAhl}$ is used in the Westcott formalism based calculation. So the value $\phi_{th}$ can be safely used in placement of $\phi_{West\;\cot t}$. This is agreed with calculation performed by other authors [5]. 

The k-factor in eq. N.1 is, however, generated from any values of Westcott’s factor *g*(*T_n_*), so eq. (N.2) can be re-written as follows: (N.3)
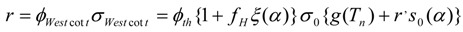


Because $(\phi_{West\;\cot\;t}/\phi_{th,HogdAhl})=\{1+f_{H}\xi (\alpha )\}≅1$ as mentioned above, the reaction rate for the “*non* − 1/*v_n_*” - named (n,γ) reactions can be calculated as: (N.4)
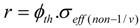

(N.5)
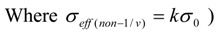


For the “1/*v_n_*”- named (n,γ) reactions, the reaction rate in both thermal and epithermal neutron region calculated based on the Hogdahl convention ion is: (N.6)
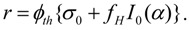

with $\sigma_{eff(1/v)}=\sigma_{0}+f_{H}I_{0}(\alpha )$ it is written as:(N.7)
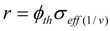


For the above equations, $\phi_{th,HogdAhl}$ or $\phi_{th}$ is Hogdahl convention thermal neutron flux, $f_{H}=\phi_{epi}/\phi_{th}$ is so-called Hogdahl conventional neutron flux ratio or epithermal to thermal (subcadmium) neutron flux ratio ( in this paper *R_epi_* is used instead *i.e f_H_* = *R_epi_*). *Q*_0_ = *I*_0_/*σ*_0_ is infinite dilution resonance integral *I*_0_ per thermal neutron activation cross-section *σ*_0_ at the corresponding energy *E*_0_ = 0.0253*eV*; $I_{0}=\int_{Ec}^{1\;MeV}\frac{\sigma (E)}{E}dE;\;I_{0}(\alpha )=Q_{0}(\alpha )\sigma_{0}:$

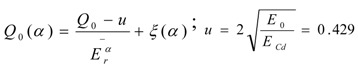


$\xi (\alpha )\;=\;0.429/\{(1+2\alpha )E_{Cd}^{\alpha }\}$ is for the cadmium cut-off energy *E_Cd_* = 0.55 *eV* correction,

*α* is epithermal flux distribution parameter, its extreme values −0.15 < *α* < +0.3, 

*σ_eff_*_(1/*v*)_ is Hogdahl convention effective cross-section. *n_n_* is total neutron density.

*v_n_* is neutron velocity and *v_0_* is the most probable neutron velocity at 20 °C (2200 m/s).

*g*(*T_n_*) is Westcott’s g-factor for neutron temperature *T_n_*, *g*(*T_n_*) ≠ 1 for “*non* − 1/*v_n_*” reactions.

*r* is a measure for the epithermal to total neutron density ratio in the Westcott formalism, r=r(α)TnT0⋅S0(α)

*S*_0_ is ratio of the modified reduced resonance integral (1/*v_n_* − tail subtracted) to the thermal cross-section *σ*_0_, 

$S_{0}=\frac{2}{\sqrt{\pi }}\{Q_{0}-u\}$ and $S_{0}(\alpha )=\frac{2}{\sqrt{\pi }}\{Q_{0}(\alpha )-\xi (\alpha )\}$

$\phi_{West\;\cot t}$ is Westcott conventional (total) neutron flux,

$\sigma_{West\;\cot t}$ is Westcott convention effective cross-section,

$\sigma_{eff(non-1/v)}$ is “*non* − 1/*v_n_*” effective cross-section,

## 3. Experimental

### 3.1. Reagents and materials

The isotopically enriched ^176^Yb_2_O_3_ and ^176^Lu_2_O_3_ targets for neutron activation were purchased from Trace-Sciences International Inc.USA [10]. The ^176^Yb_2_O_3_ target isotopic compositions were ^176^Yb (97.6%), ^174^Yb (1.93%), ^173^Yb (0.18%), ^172^Yb (0.22%), ^171^Yb (0.07%), ^170^Yb (<0.01%) and ^168^Yb (<0.01%). The main Lanthanide impurities of this target were Er (50 p.p.m), Tm (50 p.p.m) and Lu (50 p.p.m). The ^176^Lu_2_O_3_ target isotopic compositions were ^176^Lu (74.1 %), ^175^Lu (25.9%). The main Lanthanide impurities of this target were La (66 p.p.m), Yb (13 p.pm), Tm (<1 p.p.m), Er (17 p.p.m), Dy (4 p.p.m), Gd (6 p.p.m), Eu (20 p.p.m), Sm (2 p.p.m) and Nd (1 p.p.m).

### 3.2. Targets, reactor irradiations, chemical separation, elemental analysis and radioactivity calibration

The radioactive ^177^Lu + ^175^Yb solutions were obtained by the reactor thermal neutron irradiation of ^176^Yb_2_O_3_ and/or ^176^Lu_2_O_3_ targets. A quartz ampoule containing an adequate amount of ^176^Yb_2_O_3_ or ^176^Lu_2_O_3_ target was irradiated with a thermal neutron flux in HIFAR reactor (Australia). A 24-hour cooling period was needed to let all ^177^Yb (T_1/2_ = 1.911 hours) radionuclides (which formed via ^176^Yb (n, γ) ^177^Yb) to be transformed to ^177^Lu via beta particle decay. The irradiated target was then dissolved in HCl solution and the radiochemical separation of ^177^Lu from the target solution was performed as reported in our previous papers [8,9]. The radioactivity of the different radioisotopes was calibrated using a CAPINTEC Dose calibrator and gamma-ray spectrometer coupled with ORTEC HP Ge detector. The gamma ray energy and counting efficiency calibration of this analyzer system were performed using a radioactive standard source of ^152^Eu solution. Lutetium element and other metal content in the completely decayed ^177^Lu solutions (at least > 10 half-lives) was analyzed using ICP-MS instrument. 

## 4. Results and Discussion

The methods developed in the above sections were evaluated and used for the assessment of SA values of two typical isotope target systems, enriched ^176^Lu and ^176^Yb targets. ^177^Lu produced from these targets is a representative for the state-of-the-art radioisotopes of high specific radioactivity used in targeted endo-radiotherapy.

### 4.1. The specific radioactivity of ^177^Lu radioisotope produced via ^176^Lu (n, γ)^177^Lu reaction

The ^176^Lu enriched target is used for ^177^Lu production. The main nuclear characteristics and nuclear reactions/ radioactive transformations of the ^176^Lu and ^175^Lu isotope are listed in Table 1. The production of ^177^Lu radioisotope is based on the reaction Lu-1. As shown in this data list, the target composes of two stable isotopes, ^176^Lu and ^175^Lu. In the reactions Lu-6 and Lu-3 the neutron captures yield the isotopes of another chemical element, so these reactions may cause a depression in elemental Lu atom numbers of the target during neutron bombardment.

However, the effect of these reactions is ignored due to their low cross sections. The reactions Lu-2, Lu-4 and Lu-5 yield long –lived and stable isotopes of the Lutetium element, so the elemental Lu atom numbers of these isotopes are likely to be unchanged during neutron irradiation. This condition shows a similarity between the ^176^Lu enriched target and the multi-isotope target system of depression factor Δ_*S*_2,*A*__ = 0 as described in Section 2.1.2.2 (Third bullet). So, eqs. 20–25 are adopted for the ^177^Lu specific radioactivity assessment of the Lu target. For this SA assessment process the relevant parameters should be identified to individualize the selected equations for the above mentioned ^177^Lu production reaction. These parameters are the following:

S_1,A_ is ^176^Lu and re-denoted as S_1,Lu_. S_2,A_ is ^175^Lu and re-denoted as S_2,Lu_. R_i_ is ^177^Lu.

P_1_ is the weight percentage of ^176^Lu. P_imp,A_ is weight percentage of ^175^Lu and re-denoted as P_imp,Lu_ . M_1_ is atomic weight of ^176^Lu. M_imp,A_ is atomic weight of ^175^Lu and re-denoted as M_imp,Lu_. $\Delta_{S_{1,A}}$ is $\Delta_{S_{1,Lu}}$ for isotope ^176^Lu.

Because ^176^Lu has three neutron capture reactions (i=1, 2, 3) as listed in Tab. 1, the $\Delta_{S_{1,Lu}}$ value is $\Delta_{S_{1,Lu}}=\phi_{th}\cdot \sum_{i=1}^{i=3}\Omega_{1,i}\;\mathrm{or}\;\Delta_{Lu-176}=\phi_{th}\cdot \sum_{}^{}\left(\sigma_{1,i(th)}+R.\sigma_{1,i(res)}\right),$

where $\Omega_{1,i}=\sigma_{1,i(th)}+R_{epi}\cdot \sigma_{1,i(epi)}+R_{fAst}\cdot \sigma_{1,i(fAst)}$ or $\Omega_{1,1}=\sigma_{1,1(th)}+R\cdot \sigma_{1,1(res)}$ or $\Omega_{1,Lu-177}=\sigma_{1,Lu-177(th)}+R\cdot \sigma_{1,Lu-177(res)}$

Because the ^176^Lu is a typical non-1/υ_n_ nuclide, the *σ*_1,*i*(*th*)_ cross section (of reaction Lu-1 in Table 1), which is tabulated for neutron of E= 0.0253 eV and υ_n_ = 2,200 m/s, should be multiplied with a so called k-factor which is based on the Westcott convention equation as discussed in Section 2.3 [5,6]. The k values ranging from 1.67 at 10 °C to 1.9 at 40 °C were calculated for the Munich reactor [5]. As generally accepted, we use the value k = 1.74 tabulated in reference [11] for our further calculation. 

$\Omega_{1,i}=\sigma_{1,i(th)}+R_{epi}\cdot \sigma_{1,i(epi)}+R_{fAst}\cdot \sigma_{1,i(fAst)}=\sigma_{eff(non-1/v)}+R_{fAst}\cdot \sigma_{1,i(fAst)}=k\cdot \sigma_{0}+R_{fAst}\cdot \sigma_{1,i(fAst)}$or $\Omega_{1,i}=1.74\cdot \sigma_{0}+R_{fAst}\cdot \sigma_{1,i(fAst)}$

(the item $R_{fAst}\cdot \sigma_{1,i(fAst)}$ can be ignored due to the insignificant value of fast neutron flux)

$\Delta_{S_{2,A}}$ is $\Delta_{S_{2,Lu}}$ for isotope ^175^Lu and $\Delta_{S_{2,Lu}}=\phi_{th}\cdot \sum_{y=1}^{y=3}\Omega_{2,y}\;=0\;$

where $\Omega_{2,y}=\sigma_{2,y(th)}+R_{epi}\cdot \sigma_{2,y(epi)}+R_{fAst}\cdot \sigma_{2,y(fAst)}=0$

$\Lambda_{R_{i}}$ is $\Lambda_{Lu-177}$ for radionuclide ^177^Lu and $\Lambda_{Lu-177}=\lambda_{Lu-177}+\Delta_{Lu-177}$

where $\Delta_{Lu-177}=\phi_{th}\cdot \sum \Omega_{Lu-177}$ and $\lambda_{Lu-177}=\sum_{m=1}^{m=j}\lambda_{m,Lu-177}$

**Figure 1:**
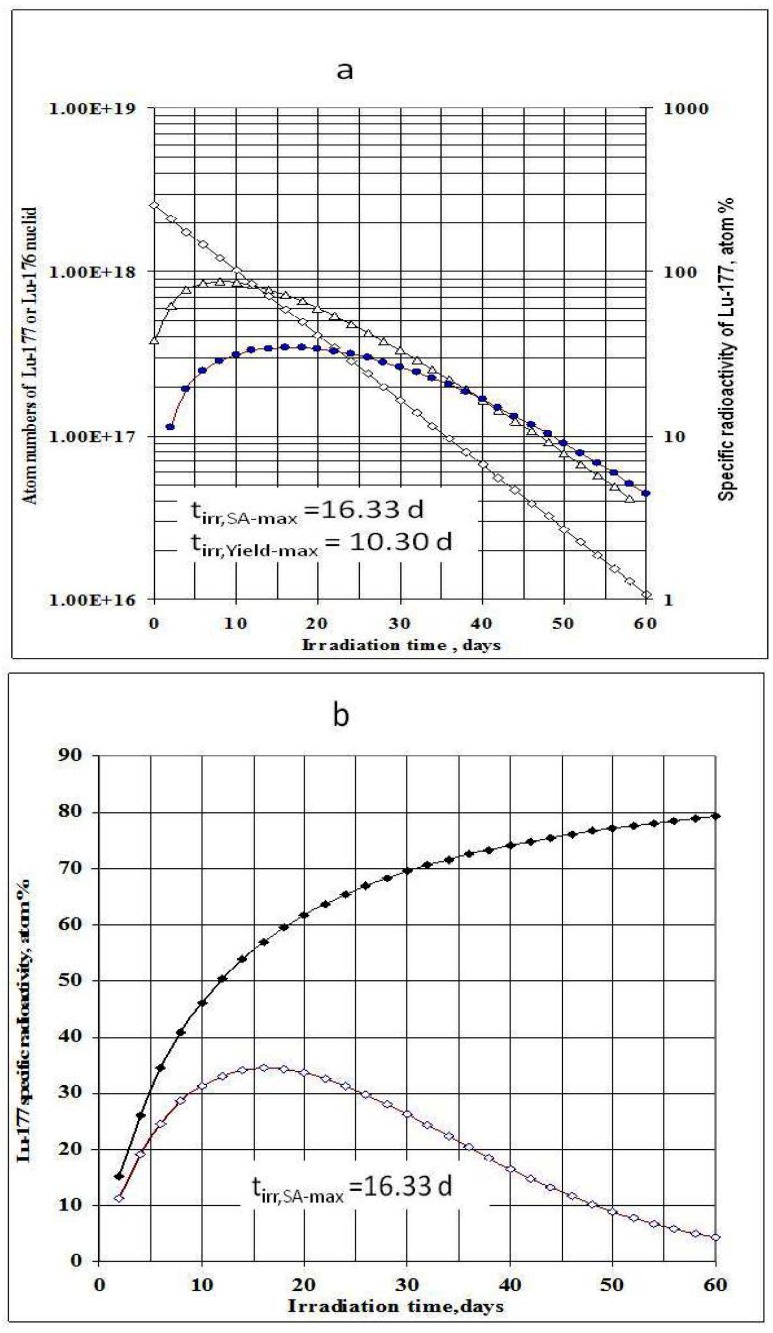
**a-** ^176^Lu target nuclide depression, ^177^Lu build-up and ^177^Lu specific radioactivity in the ^176^Lu enriched target *vs*. irradiation time (Thermal neutron flux: 2.5·10^14^ *n*·*cm*^−2^·*s*^−1^; Target composition: 74.1% ^176^Lu + 25.9% ^175^Lu; Target weight: 1.0 mg), •−•−•: Specific radioactivity of ^177^Lu; Δ–Δ–Δ: Atom numbers of ^177^Lu; ◊-◊-◊: Atom numbers ^176^Lu target nuclide. **b-** ^177^Lu specific radioactivity in the ^176^Lu enriched target vs. irradiation time and ^176^Lu enrichment of the target (Thermal neutron flux: 2.5·10^14^ *n*·*cm*^−2^·*s*^−1^), ♦-♦-♦: 100% purity ^176^Lu target; ◊-◊-◊: Target composition: 74.1% ^176^Lu + 25.9 % ^175^Lu.

**Figure 2:**
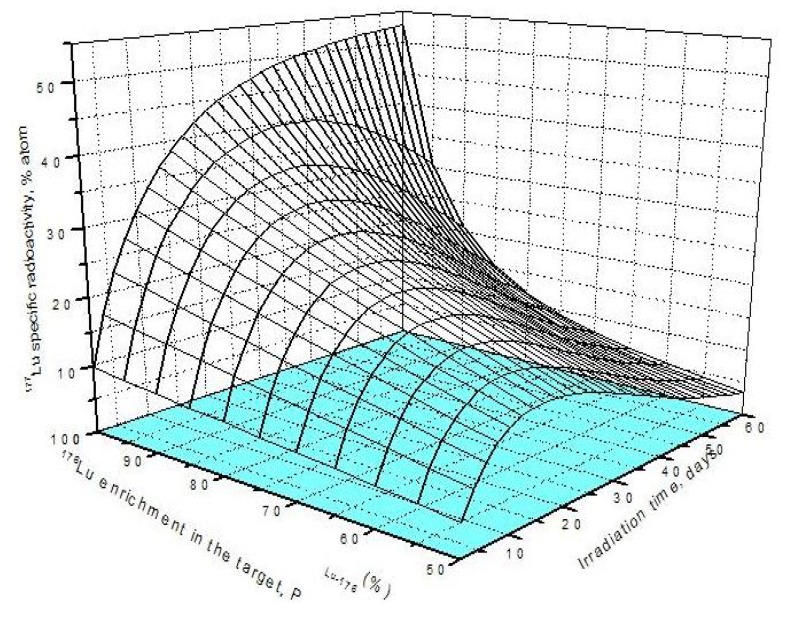
^177^Lu specific radioactivity as a function of irradiation time and ^176^Lu isotopic purity in the target (Thermal neutron flux of 1.7·10^14^ *n*·*cm*^−2^·*s*^−1^ was applied. Nuclear data was extracted from literatures [11]).

^177^Lu specific radioactivities as a function of the target isotopic composition, neutron flux and irradiation time were formulated and calculated (as shown in Figure 1 and Figure 2). The maximum values of ^177^Lu specific radioactivity and irradiation time were evaluated. These were used as optimal conditions for carrier-containing ^177^Lu production.

**Figure 3:**
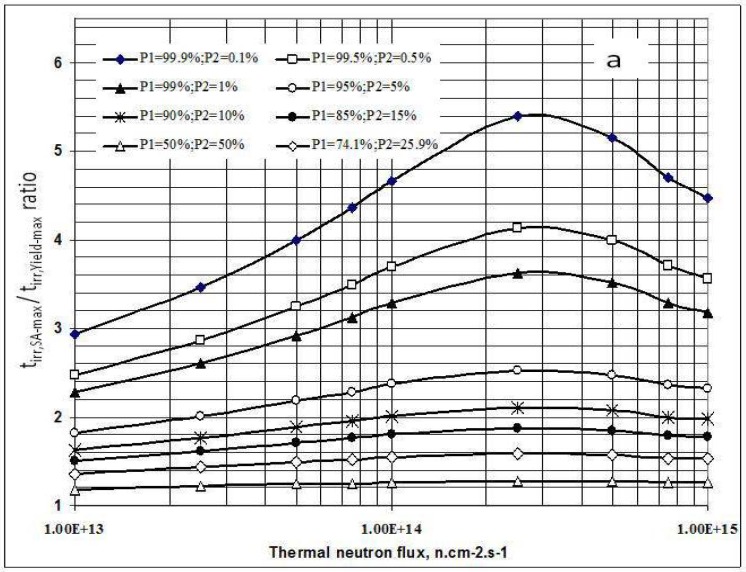

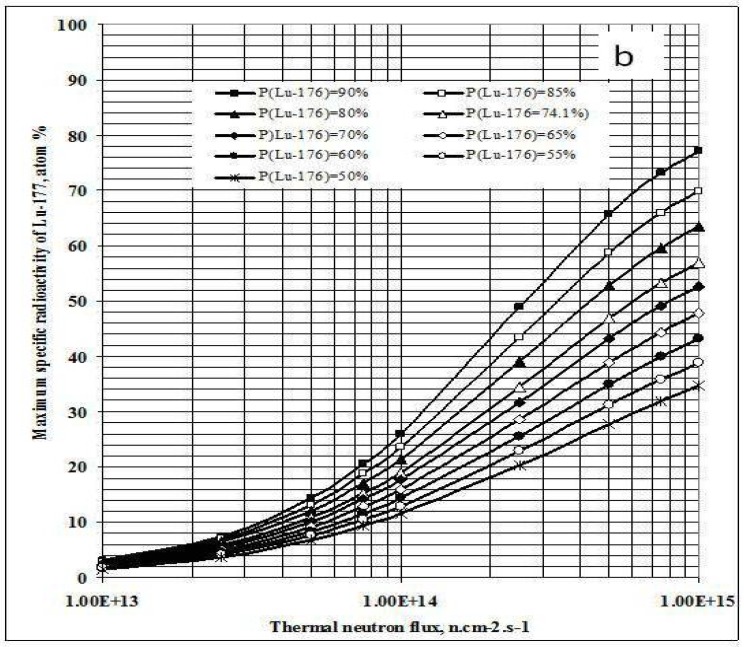
**a**- Irradiation time ratio (t_irr,SA-max_ / t_irr,Yield-max_) *vs*. thermal neutron flux and target composition.**b**- Maximum specific radioactivity of ^177^Lu *vs*. thermal neutron flux and target composition.

As shown in Figure 1a, the irradiation time for maximum yield (t_irr,Yield-max_) and that for maximum specific radioactivity (t_irr,SA-max_) are different. The results presented in Figure 3a state that the higher the ^176^Lu enrichment of the target, the bigger the difference between values t_irr,Yield-max_ and t_irr,SA-max_. The ratio of these times varies with thermal neutron flux applied and reaches a maximum value at the flux value of around 3·10^14^ *n*·*cm*^−2^·*s*^−1^ for all the ^176^Lu enrichment values of the target.

This result is quite interesting in respect to the optimization of neutron flux utilization, irradiation time and target enrichment. Based on the result of further analysis of eqs. (15) and (24) the conclusion can be drawn that the coincident interaction based on the target burn-up / product depression ratio (Δ_*S*_1,*A*__/Λ*_R_i__*) plays an important role in the creation of this maximum value of t_irr,Yield-max_ and t_irr,SA-max_ / t_irr,Yield-max_ ratio at a specified neutron flux value specific for a given target system.. Although the higher neutron flux irradiation gives the higher SA as shown in Figure 3b, the bigger difference between values t_irr,Yield-max_ and t_irr,SA-max._ makes the outcomes of maximum yield and maximum SA incompatible. 

This is to say that the production of ^177^Lu via ^176^Lu (n,γ) ^177^Lu reaction with neutron flux of around 3·10^14^ *n*·*cm*^−2^·*s*^−1^ could be awkward. Hence the production yield and desired SA should be compromised to achieve a cost effective production of clinically useful ^177^Lu product. The t_irr,SA-max_ values increase with the ^176^Lu enrichment on the target (Figure 2) and the 100% purity ^176^Lu target showed no-maximum SA value during neutron activation as seen in Figure 1b. This is confirmed by the analysis of differential equation 27, which was described in Section 2.1.2.3 above.

### 4.2. The specific radioactivity of ^177^Lu radioisotope produced via neutron- capture- followed- by- radioactive transformation, ^176^Yb (n, *γ*) ^177^Yb (*β*^-^ decay) ^177^Lu

The ^176^Yb enriched target is used for ^177^Lu radioisotope production. Based on the isotopic compositions of the elemental Lu -contaminated ^176^Yb_2_O_3_ target and the possible nuclear reactions listed in Table 1 and Table 2, the total ^177^Lu radioactivity in this activated ^176^Yb target composes of one part (denoted as *A*_1-*Lu*-177_) induced from the ^176^Yb target nuclide via reaction^176^Yb (n, *γ*) ^177^Yb (*β*^-^ decay) ^177^Lu and another part (denoted as *A*_2-*Lu*-177_) from the^176^Lu impurity via ^176^Lu (*n*, *γ*) ^177^Lu reaction. As shown in Table 2, the Lu-free ^176^Yb target contains ^174^Yb impure isotope, so the ^175^Lu induced via reaction^174^Yb (n, *γ*) ^175^Yb (*β*^-^ decay) ^175^Lu increases the atom numbers of elemental Lu in the target during neutron activation. Especially, due to long-lived^175^Yb, the ^175^Lu generation from beta decay of ^175^Yb will be continued during post-irradiation processing/cooling of the target. The SA degradation effect of ^174^Yb and Lu impurities on the carrier-free ^177^Lu producible from an isotopically pure^176^Yb target is demonstrably predictable. The assessment of this effect measure is a showcase example for a complex target system in which a stable brother isotope of radioisotope R_i_ may be generated from the elemental impurities.

Specific radioactivity SA_Lu-177_ achievable in the Lu- and ^174^Yb-contaminated ^176^Yb target will be calculated based on “isotopic dilution” equation (35) which is applied for a multi radioactive source system. The ^176^Yb enriched target discussed in this report is referred to as a system composed of two ^177^Lu radioactive sources, S_1_ and S_2_. The source S_1_ refers to the radioisotopes induced in the elementally pure (Lu impurity-free) Yb target, while S_2_ is the radioactive part produced from the Lu impurity in the target. 

#### 4.2.1. ^177^Lu radioactive source 1 (S_1_): Radioactivity (A_1-Lu--177_) and specific radioactivity (*SA*_1-*Lu*--177_) of ^177^Lu isotope in the elementally pure (Lu impurity-free) Yb target

As shown in Table 2, the target is composed of seven stable Yb isotopes. Among them only ^176^Yb and ^174^Yb are involved in the neutron capture reactions (reactions Yb-1 and Yb-2, respectively) to produce the Lu isotopes (^177^Lu and ^175^Lu). It is obvious that in the elementally pure (Lu impurity-free) Yb target, the ^177^Lu radioactivity is generated from reaction Yb-1. The elemental Lu atom numbers, however, result from both reactions Yb-1 and Yb-2. The following parameters should be evaluated for the SA assessment of ^177^Lu isotope in the given Yb target.

Radioactivity of carrier-free ^177^Lu ( A_1-Lu—177_ ) from the reaction Yb-1 in the Lu -free Yb target.

Radioactivity at post-irradiation time t_c_ (*A*_1-*Lu*-177,*t_c_*_) can be calculated using eq.28. The carrier-free ^177^Lu activity is the following: (36)
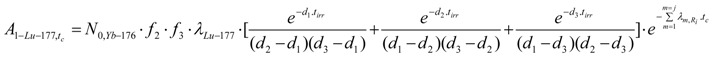


The relevant parameters should be identified to individualize this equation for the reaction Yb-1 mentioned in Table 2. Individualizing eq.28 with Ω_1,1_ for Ω*_Yb_*_-176,1_ (or Ω*_Yb_*_-176,*Yb*-177_),Ω_x_ for Ω*_Yb_*_-177_ and Ω*_i_* for Ω*_Lu_*_-177_, these parameters are the following:

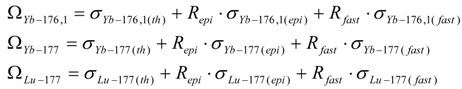

(*R_fast_*·*σ_Yb_*_-176,1(*fast*)_, *R_fast_*·*σ_Yb_*_-177(*fast*)_and *R_fast_*·*σ_Lu_*_-177(*fast*)_ can be ignored due to the insignificant value of fast neutron flux) 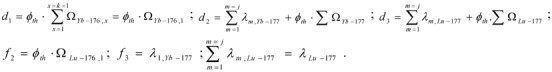

N_0,Yb-176_ denotes the atom numbers of ^176^Yb in the m grams weight target.

*N*_0,*Yb*-176_=6.02·10^23^·*m*·*P_Yb_*_-176_/(100·176), where P_Yb-176_ is the weight percentage of ^176^Yb in the ^176^Yb enriched target. 

*Total elemental Lu atom numbers* N_1-Lu_ *in the Lu impurity-free ^176^Yb target.* This amount is the sum of the Lu atom numbers of carrier-free ^177^Lu radioisotope (*N*_1-*Lu*-177,*t_c_*_) from reaction Yb-1 and Lu atom numbers generated from the ^174^Yb impurity of the target (*N*_1-*Lu*-175,*t_c_*_) from reaction Yb-2.(37)
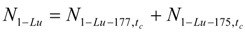


*N*_1-*Lu*-177,*t_c_*_ is calculated by a quotient of eq.36 and *λ_Lu_*_-177_: (38)
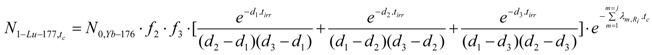


*N*_1-*Lu*-177,*t_c_*_ calculation is adopted from eq.33b described in Section 2.2.1.2.(39)
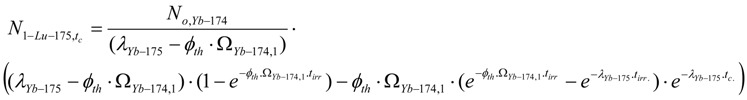


Individualizing eq. (33b) for the ^175^Lu isotope generated from ^174^Yb, the following is identified. 

S_2,B_ is stable isotope^174^Yb. $\Delta_{S_{2,B}}$ is re-denoted as $\Delta_{Yb-174}$ for isotope ^174^Yb. Because ^174^Yb has only one neutron capture reaction (y = 1) as shown in Tab. 2, the $\Delta_{Yb-174}$ value is $\Delta_{Yb-174}=\phi_{th}\cdot \sum_{y=1}^{y=1}\Omega_{Yb-174,y}=\phi_{th}\cdot \Omega_{Yb-174,1}$, where $\Omega_{Yb-174,y}=\sigma_{Yb-174,y(th)}+R_{epi}\cdot \sigma_{Yb-174,y(epi)}+R_{fast}\cdot \sigma_{Yb-174,y(fast)}$. (The items $R_{fast}\cdot \sigma_{Yb-174,y(fast)}$ can be ignored due to the insignificant value of fast neutron flux).

$\Delta_{R_{y}}$ is re-denoted as $\Delta_{Yb-175}$ for radioisotope ^175^Yb and $\Delta_{Yb-175}=\phi_{th}\cdot \sum \Omega_{Yb=175}$. 

$\Lambda_{R_{y}}$ is re-denoted as $\Lambda_{Yb-175}$, $\sum_{m=1}^{m=j}\lambda_{m,R_{y}}$ as $\sum_{m=1}^{m=j}\lambda_{m,Yb-175}$ and $\lambda_{R_{y}}\to S_{g,A}$ as $\lambda_{Yb-175\to Lu-175}$ for radionuclide Yb_175_ and $\Lambda_{Yb-175}=\sum_{m=1}^{m=j}\lambda_{m,Yb-175}+\Delta_{Yb-175}$. For simplification, $\sum_{m=1}^{m=j}\lambda_{m,Yb-175}=\lambda_{Yb-175\to Lu-175}$ is applied and value $\Delta_{Yb-175}$ is ignored due to unavailability of $\sum \Omega_{Yb-175}$ value. So we get $\Lambda_{Yb-175}=\lambda_{Yb-175\to Lu-175}=\lambda_{Yb-175}$. $N_{0,S_{2,B}}$ is the initial ^174^Yb atom numbers $N_{0,Yb-174}$. This quantity is $N_{0,Yb-174}=6.02\cdot 10^{23}\cdot m\cdot P_{Yb-174}/(100\cdot 174)$. P _Yb-174_ is the weight percentage of ^174^Yb impurity in the ^176^Y target.

*^177^Lu specific radioactivity of the Lu impurity-free ^176^Yb target*. The specific radioactivity of ^177^Lu source (the ^177^Lu radioactive source 1) generated from the elemental Lu impurity-free *^176^Yb* target is the following: (40)
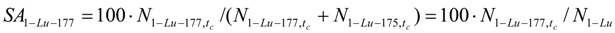


#### 4.2.2. ^177^Lu radioactive source 2 (S_2_): Radioactivity A_2-Lu-177)_ and specific radioactivity *SA*_2-*Lu*-177_ of ^177^Lu generated from ^176^Lu (*n*, *γ*) ^177^Lu reaction of the elemental Lu-impurity in the Lu-contaminated ^176^Yb target 

*Specific radioactivity*. *SA*_2-*Lu*-177_ of ^177^Lu radioisotope produced in this target is calculated using the same process as described in the previous Section 4.1 ‘The specific radioactivity of ^177^Lu radioisotope produced via ^176^Lu (n, *γ*)^177^Lu reaction’ of Results and Discussion. In this case the impure Lu content in the ^176^Yb target is assumed to have natural abundance, so the values P_1_ = 2.59% for ^176^Lu and P_imp,Lu-175_ = 97.41% for ^175^Lu are put into calculation.

*Radioactivity.* Radioactivity of ^177^Lu from the elemental Lu impurity in the ^176^Yb target at post-irradiation time t_c_ is calculated using eq. (14): (41)
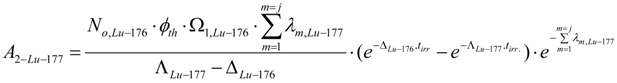

$N_{0,Yb-176}=6.02\cdot 10^{23}\cdot m\cdot P_{1}/(100\cdot 176);\;$ P _1_ =2.59 % is natural abundance of ^176^Lu. m is the weight of elemental Lu impurity in the ^176^Y target. 

#### 4.2.3. ^177^Lu specific radioactivity *SA_Lu_*_-177_ and ^177^Lu radioactivity *A_Lu_*_-177_ in the Lu-contaminated ^176^Yb target as a combination of ^177^Lu radioactive source S_1_ and S_2_

*^177^Lu specific radioactivity SA_Lu_*_-177_. The method of SA assessment of the mixture of several radioactive sources (referred to Section 2.2.2) is used for the SA calculation. The ^177^Lu specific radioactivity *SA_Lu_*_-177_ in the Lu-contaminated ^176^Yb target is calculated using eq. (35) with relevant parameters of the mixture of two ^177^Lu radioactive sources S_1_ and S_2_ as described above,(42)
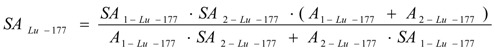


*^177^Lu radioactivity.* Total ^177^Lu radioactivity in the target is *A_Lu_*_-177_ = *A*_1*-Lu*-177_ + *A*_2*-Lu*-177_. ^175^Lu isotope generated from ^174^Yb (n, *γ*) ^175^Yb (*β*^-^ decay) ^175^Lu process and elemental Lu impurities remaining in the ^177^Lu product (which is chemically separated from ^176^Yb target) makes the SA value of ^177^Lu strongly decreased. 

Rendering the differential of eq. (42) equal to zero offers the way to calculate the irradiation time at which the SA of ^177^Lu reaches maximum value (*SA_Lu_*_-177,max_). The maximum SA is predicted based on the opposite tendency of SA variation of elementally pure and Lu-contaminated Yb targets. This argument is supported by the results obtained below.

**Figure 4:**
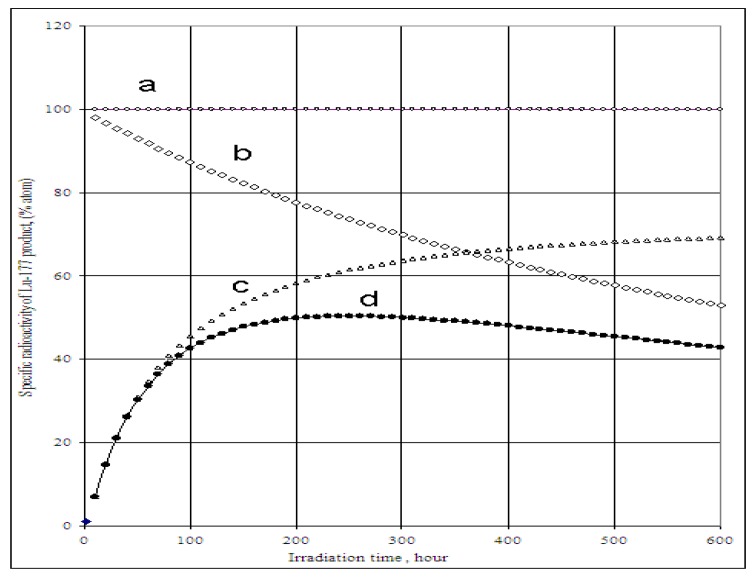
Specific radioactivity of ^177^Lu radioisotope in the ^176^ Yb target vs. irradiation time and content of ^174^Yb- and elemental Lu- impurities.(Thermal neutron flux: 5·10^13^ *n*·*cm*^−2^·*s*^−1^, Nuclear data extracted from literatures [11]).

**Figure 5:**
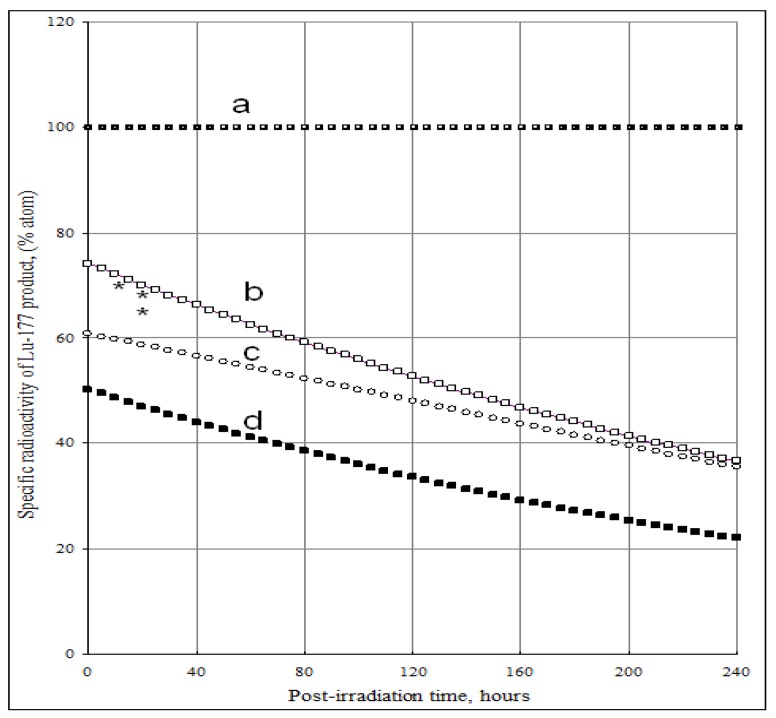
Specific radioactivity of ^177^Lu in the ^176^ Yb target vs. post-irradiation time and content of ^174^Yb and elemental Lu impurities. (Thermal neutron flux: 5·10^13^ *n*·*cm*^−2^·*s*^−1^; Irradiation time: 240 hours; Nuclear data extracted from literatures [11]).

The SA of ^177^Lu radioisotope produced by the ^176^Yb (n, *γ*) ^177^Yb (*β*^-^ decay) ^177^Lu process as a function of the elemental and isotopic impurities of the ^176^Yb enriched target and the times t_irr_ and t_c_ is shown in Figure 4 and Figure 5. The experimental results reported in our previous publications agree well with the theoretical calculation results shown in Figure 5. The maximum SA value present on the curve D of Figure 4 shows a combined effect of ^174^Yb- and elemental Lu- impurities on the SA degradation. This tells us that the irradiation time should be optimized to obtain the highest SA for ^177^Lu produced via ^176^Yb (n, *γ*) ^177^Yb (*β*^-^ decay) ^177^Lu reaction. While being the best theoretical way to produce carrier-free ^177^Lu, with this reaction we always obtain a ^177^Lu product of much lower SA due to the use of an isotopically/elementally impure target. The maximum SA value mentioned above is characterized for a specific target composition and its neutron irradiation conditions, so the theoretical assessment of SA developed in this paper is important before starting the neutron activation process. This avoids over-bombardment destroying the SA of ^177^Lu product and wasting expensive reactor operation time. Moreover, the post-irradiation processing time should be minimized to keep the SA as high as possible, the effect of which is shown in Figure 5.

## 5. Conclusions

Several factors affect SA of the radionuclide product which can be produced either by neutron capture reaction or by neutron-capture-followed- by -radioactive transformation processes. Among them the target composition (elemental and isotopic impurities), target nuclide and produced radioisotope depression causes (including target nuclide burn-up, reaction rate of target nuclide and the decay property of produced radionuclide) and the activation or post-irradiation time are most accounted for. With the method of SA assessment of multi radioactive source system the SA of a radioisotope produced in a reactor from different targets can be evaluated. The theoretical SA assessment of a radioactive nuclide has definitely given us a firm basis to set up an optimized process for the production of clinically useful radioisotopes and to evaluate the quality of the radionuclide product. A useful computer code based on the above developed SA assessment methods can be set up for a convenient daily use in the reactor-based production of medical radioisotopes such as ^177^Lu, ^153^Sm, ^169^Yb, ^165^Dy, ^153^Gd... This evaluation plays a complementary or even substantial role in the quality management system regarding certifying the SA of the product which may be experimentally inaccessible due to radiation protection and instrumentation difficulties in the practical measurement of very low elemental content (<0.01 μg/mCi·μL) of a radioactive solution of very high specific volume and specific radioactivity. 

## Figures and Tables

a: Specific radioactivity of ^177^Lu isotope in impurity-free ^176^Yb target; b: Specific radioactivity of ^177^Lu isotope in the ^176^Yb target containing 1.93% ^174^Yb; c: Specific radioactivity of ^177^Lu isotope in the ^176^Yb target containing 50 p.p.m Lu element impurities; d: Specific radioactivity of ^177^Lu isotope in the ^176^Yb target containing 1.93% ^174^Yb and 50 p.p.m Lu element impurity.

a: SA of ^177^Lu isotope in the impurity-free ^176^Yb target; b: SA of ^177^Lu isotope in the ^176^Yb target containing 1.93% ^174^Yb; c: SA of ^177^Lu isotope in the ^176^Yb target containing 50 p.p.m Lu element impurities; d: SA of ^177^Lu isotope in the target containing 1.93% ^174^Yb and 50 p.p.m Lu element impurity. (*) is the experimental measurement result for this type of target.

**Table 1:** Nuclear characteristics of the radionuclides produced in the ^176^Lu enriched target [11].

Target Stable Isotope (Denoted)	Conc. in target	Cross Sections, Barn		Nuclear reaction and product (T_1/2_)	Reaction No. (Reaction branch)
		σ_th or_ σ_0_	σ_epi or_ I_0_		
^176^Lu (S_1,Lu_)	P_1_ =74.1 %	2300	1200	^176^Lu (n,γ)^177^Lu (6.7d)^(*)^ $\underset{\to }{\beta –177}$Hf (stable)	Lu-1 (i = 1)
		2	3	^176^Lu (n,γ)^177m^Lu(160.7d) $\underset{\to }{\mathrm{IT}–177}$Lu(6.7d) $\underset{\to }{\beta –177}$Hf(s)	Lu-2 (i = 2)
		<2.10^−3^	-	^176^Lu (n,α) ^173^Tm (8.2h) $\underset{\to }{\beta –173}$Yb ( Stable)	Lu-3 (i = 3)
^175^Lu(S_2,Lu_)	P_imp,Lu_ =25.9 %	16	550	^175^Lu (n,γ) ^176m^Lu (3.7h) $\underset{\to }{\mathrm{IT}–176}$Lu ( Stable)	Lu-4 (y = 1)
		9	300	^175^Lu (n,γ) ^176^Lu ( Stable)	Lu-5 (y = 2)
		<10^−5^		^175^Lu (n,α) ^172^Tm (2.6d ) $\underset{\to }{\beta –172}$Yb (Stable)	Lu-6 (y = 3)

* ^177^Lu depression caused by possible (n, γ) and/or (n, p) reactions is ignored compared to radioactive decay rate of ^177^Lu isotope.

**Table 2:** Nuclear characteristics of radionuclides produced in ^176^Yb target matrix [11].

Stable Isotope (Denoted)	Conc. in target(%)	Cross Section, Barns		Nuclear reactions and products (T_1/2_)	Reaction No. (Reaction branch)
		σ_th or_ σ_o_	σ_epi or Io_		
^176^Yb (S_1,B_)	97.6	3.0	8	^176^Yb(n,γ) ^177^Yb (1.9h) $\underset{\to }{\beta –177}$Lu (6.7d) $\underset{\to }{\beta –177}$Hf (stable)	Yb-1 (x = 1)
^174^Yb (S_2,B_)	1.93	63.0	60	^174^Yb (n,γ) ^175^Yb (4.2d) $\underset{\to }{\beta –175}$Lu (Stable)	Yb-2 (y = 1)
^173^Yb (S_3,B_)	0.18	17.4	400	^173^Yb (n,γ) ^174^Yb (Stable)	Yb-3 (z = 1)
^172^Yb (S_4,B_)	0.22	1.3	25	^172^Yb (n,γ) ^173^Yb (Stable)	Yb-4 (p = 1)
^171^Yb (S_5,B_)	0.07	50.0	320	^171^Yb (n,γ) ^172^Yb (Stable)	Yb-5 (q = 1)
^170^Yb (S_6,B_)	<0.01	10.0	300	^170^Yb (n,γ) ^171^Yb (Stable)	Yb-6 (v = 1)
^168^Yb (S_7,B_)	<0.01	2300	2100	^168^Yb(n,γ) ^169^Yb (32d) $\underset{\to }{(\mathrm{EC})169}$Tm (Stable)	Yb-7 (w = 1)
Lu ^(*)^	5.10^-3^	Nuclear reactions and characteristics referred to Table 1			

* Elemental Lu content being of natural isotopic abundance in ^176^Yb target is assumed, i.e. 97.41 % ^175^Lu and 2.59 % ^176^Lu.
